# Expression of extracellular matrix components in the meibomian gland

**DOI:** 10.3389/fmed.2022.981610

**Published:** 2022-09-06

**Authors:** Di Chen, Xiaomin Chen, Hua-Tao Xie, Mark P. Hatton, Xiaowei Liu, Yang Liu

**Affiliations:** ^1^Department of Ophthalmology, Peking Union Medical College Hospital, Chinese Academy of Medical Sciences and Peking Union Medical College, Beijing, China; ^2^Department of Ophthalmology, Zhongnan Hospital, Wuhan University, Wuhan, China; ^3^Department of Ophthalmology, Union Hospital, Huazhong University of Science and Technology, Wuhan, China; ^4^Ophthalmic Consultants of Boston, Boston, MA, United States

**Keywords:** meibomian gland, extracellar matrix, stem cell niche, biomarker, organ regeneration

## Abstract

**Purpose:**

Extracellular matrix (ECM) is a key component of the stem cell local microenvironment. Our study aims to explore the periglandular distribution of major components of ECM in the Meibomian gland (MG).

**Methods:**

Human eyelids and mouse eyelids were collected and processed for immunofluorescence staining.

**Results:**

Human MG tissues stained positive for collagen IV α1, collagen IV α2, collagen IV α5, and collagen IV α6 around the acini and duct, but negative for collagen IV α3 and collagen IV α4. The mouse MG were stained positive for the same collagen IV subunits as early as postnatal day 15. Laminin α2, laminin β1 and perlecan stained the regions surrounding the acini and the acinar/ductal junction in the human MG, but not the region around the duct. Tenascin-C was found specifically located at the junctions between the acini and the central ducts. Neither agrin nor endostatin was found in the human MG tissues.

**Conclusion:**

The ECM expresses specific components in different regions around the MG, which may play a role in MG stem cell regulation, renewal, and regeneration.

## Introduction

Meibomian glands (MG) are large holocrine glands located in the eyelids, which play an important role in the maintenance of ocular surface health (1–3). The tissue is composed of clusters of acini connected to a central duct *via* ductules, as grapes grow on a vine (1). Because of the nature of holocrine secretion, the meibomian gland (MG) continuously undergoes cell replacement, which relies on the dynamic activity of stem cells. If the stem cells are exhausted, new epithelial cells cannot be generated, which leads to gland atrophy and dropout (4). The loss of MG acini is frequently observed in meibomian gland dysfunction (MGD), which is the major cause of the dry eye that affects hundreds of millions of patients in the United States (1, 2, 5–7). Unfortunately, there is no known way to regenerate MG after dropout and no cure for MGD.

It is currently believed that the MG stem cells are located at the junction between ductal and acinar basal epithelia, and that they give rise to two distinct, unipotent populations of daughter progenitor cells, which generate ductal and acinar tissues (8–10). This hypothesis is consistent with our observation that the epithelial cells of acini and ducts express different biomarkers, and that the MG acinar epithelial progenitor cells can be identified by a specific marker (11). If an individual MG stem cell gives rise to distinct progenitors, there should be different signals driving them to produce either ductal or acinar daughter cells. However, the factors that regulate and determine the fate of the MG stem cells remain unknown.

In other tissues, one of the most important regulators of stem cell fate is the extracellular matrix (ECM) (12–14). The ECM is a key component of the stem cell local microenvironment (i.e., niche), and not only provides a scaffold for the tissue, but also plays a very important role in determining stem and progenitor cell fate (15). ECM closely regulates cell behaviors including proliferation, differentiation, migration, and apoptosis (16). A study found that hyaluronan, a major ECM, plays an essential role in MG and eyelid development, which indicates that ECM may be a key factor in modulating the progeny of MG stem cells, and driving them to produce either acinar or ductal epithelial cells (17). We hypothesize that the ECM surrounding the MG acini, ducts, and at the junctions between them are all different. To begin to test our hypothesis, we investigated the periglandular distribution of several major components of ECM, including collagen IV subtypes, laminins, tenascin-C, perlecan, agrin and endostatin (18–22).

## Materials and methods

### Human and mouse tissues

Discarded and deidentified human eyelid tissues were obtained within 12 h after eyelid surgeries (five women, three men; age range, 63–88 years). Preoperative examination, including subjective questionnaire, slit lamp examination, Schirmer I-test and meibomian gland expression evaluation, confirmed normal meibomian gland structure and excluded dry eye. The use of human tissues was approved by the Institutional Review Board of the Massachusetts Eye and Ear Infirmary and Schepens Eye Research Institute (SERI) and adhered to the tenets of the Declaration of Helsinki. Eyelids from C57BL/6J mice of different ages (postnatal day 2, postnatal day 15, 2 months and 15 months old) were obtained immediately after sacrifice. At least five mice in each age group were examined. All experiments with these mice were approved by the SERI Institutional Animal Care and Use Committee (IACUC) and adhere to the Association for Research in Vision and Ophthalmology Statement for the Use of Animals in Ophthalmic and Vision Research. Tissue samples were immediately frozen in optimal cutting temperature compound (OCT, Tissue-Tek, Sakura USA, Torrance, CA) and later sectioned (15 μm) with a cryostat for staining procedures.

### Immunofluorescence staining

Eyelid sections were fixed with cold methanol for 15 minutes at −20°C. Following three phosphate-buffered saline (PBS) rinses for 5 min each, samples were blocked with 2% bovine serum albumin (BSA, Sigma-Aldrich Corp., St. Louis, MO) in PBS for 60 min, and then incubated overnight at 4°C in a moist chamber with primary antibodies specific for cytokeratin 14 (K14, ab181595, 1:500, Abcam), cytokeratin 6 (K6, ab18586, 1:500, Abcam) and extracellular matrix components (Table 1). Antibodies specific for collagen IV subtypes were generously provided by Dr. Yasuko Tomono and Dr. Yoshikazu Sado from Division of Molecular and Cell Biology, Shigei Medical Research Institute, Japan (Table 1). Isotype antibodies were applied as negative controls. After three additional PBS rinses, donkey anti-rabbit (ab150075, 1:200, Abcam) or donkey anti-mouse (2492098, 1:200, EMD Millipore, Temecula, CA) or goat anti-rat (31629, 1:200, Thermo-fisher) secondary antibodies were applied for 1 h at room temperature. C57BL/6J mouse kidney sections were used as positive controls for the collagen IV antibodies, and the mouse IgG1 control antibodies with the same concentration were used as negative controls. The slides were finally mounted using ProLong Gold antifade reagent with 4',6-diamidino-2-phenylindole (DAPI; Thermo Fisher Scientific, Waltham, MA) and observed with a confocal microscope (Leica TCS SP8, Leica Microsystems, Wetzlar, Germany).

**Table 1:** List of primary antibodies used in immunolocalization of acinar and ductal cell markers and extracellular matrix components in human meibomian gland.

**Primary antibody**	**Ig class**	**Dilution**	**Origin/vendor**	**Secondary detection system**
CK14	IgG1	1:1,000	Abcam	Cy5
CK6	IgG1	1:50	Abcam	Cy3
COL4α1	IgG2a	1:5	Sado lab	FITC
COL4α2	IgG2a	1:5	Sado lab	FITC
COL4α3	IgG2a	1:5	Sado lab	FITC
COL4α4	IgG2b	1:5	Sado lab	FITC
COL4α5	IgG2a	1:5	Sado lab	FITC
COL4α6	IgG2a	1:5	Sado lab	FITC
SOX-9	IgG2a	1:50	Santa Cruz	Cy3
Perlecan	IgG2a	1:50	Santa Cruz	FITC
Laminin α2	IgG1	1:50	Santa Cruz	FITC
Laminin β1	IgG1	1:50	Santa Cruz	FITC
Agrin	IgM k	1:50	Santa Cruz	Cy3
Endostatin	IgG k	1:50	Santa Cruz	Cy3

## Results

The major components of ECM are multiple isoforms of collagen IV and different types of glycoproteins and proteoglycans (18). We used specific antibodies to identify these compositions in human meibomian gland sections. We also investigated the localization of collagen IV subtypes in the mouse meibomian gland.

### Cytokeratins

**Figure 1:**
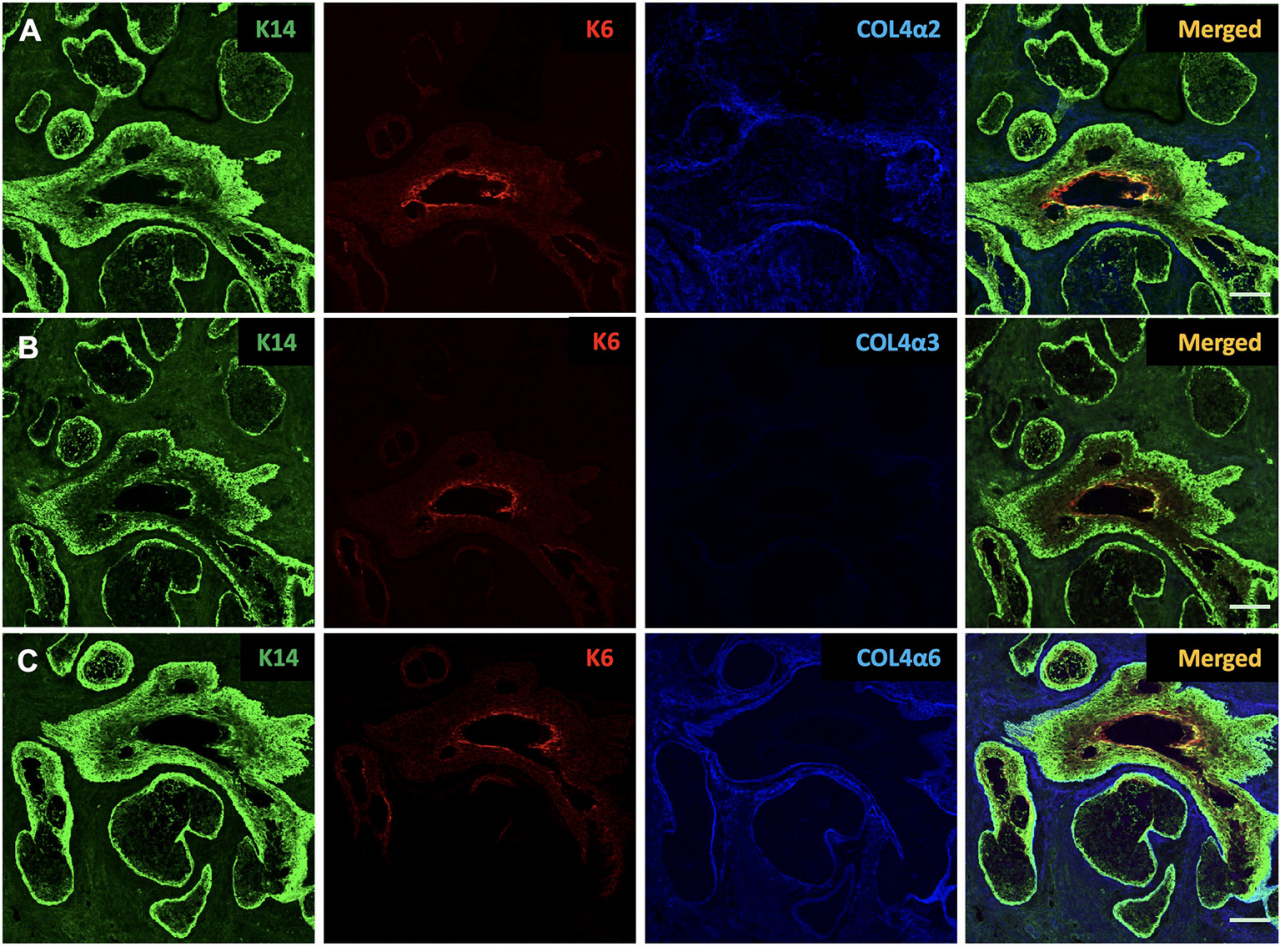
Identification of cytokeratin 14 (K14, green), cytokeratin 6 (K6, red), collagen IV α2 (COL4α2, blue), collagen IV α3 (COL4α3, blue) and collagen IV α6 (COL4α6, blue) in the human Meibomian gland (MG). K14 is present in both ductal and acinar epithelial cells, whereas K6 expression is restricted to ductal cells. Human MG tissues stain positive for COL4α2 **(A)** and COL4α6 **(C)**, but negative for COL4α3 **(B)**. Scale bar = 100 μM.

In the human tissue sections, cytokeratin 14 (K14) and cytokeratin 6 (K6) show the morphology and structure of the MG tissue (11). In Figure 1, K14 shows the structure of both the acinar and the ductal epithelia, while K6 stains only the ductal cells. The inner layer of the luminal surface of the duct shows the most intense staining for K6.

### Collagen IV subtypes

Type IV collagen is the most abundant component of basement membrane (BM), which is the major component of ECM, and comprises up to six genetically distinct α-chains, designated α1 to α6 (19). In order to test whether there are any differences in the expression of collagen IV subtypes around the MG, we used specific antibodies against collagen IV α1 (COL4α1), collagen IV α2 (COL4α2), collagen IV α3 (COL4α3), collagen IV α4 (COL4α4), collagen IV α5 (COL4α5), and collagen IV α6 (COL4α6) in our study. The specificity of these antibodies has been tested in previous publications (23–26). These α-chains are arranged into three distinct heterotrimers: α1α1α2, α3α4α5, and α5α5α6 (19). The human tissue sections were stained with all six antibodies, and the results of the staining are summarized in Table 2. Our results show that the human MG tissues stain positive for COL4α1, COL4α2, COL4α5, and COL4α6, but negative for COL4α3 and COL4α4. Figure 1 shows the human tissue sections stained with COL4α2, COL4α3, and COL4α6. These three subunits represent the localization of the distinct heterotrimers.

**Table 2:** Expression pattern of extracellular matrix components in human meibomian gland.

	**Acinar**	**Ductal**	**Junctional**
***ECM component***			
***1.Collagens***			
Type IV collagen			
α1(IV) chain	+	+	+
α2(IV) chain	+	+	+
α3(IV) chain	–	–	–
α4(IV) chain	–	–	–
α5(IV) chain	+	+	+
α6(IV) chain	+	+	+
***2. Glycoproteins***			
Laminin α2 chain	+	–	+
Laminin β1 chain	+	–	+
Tenascin-C	–	–	+
***3. Proteoglycans***			
Perlecan	+	–	+
Agrin	–	–	–
Endostatin	–	–	–

**Figure 2:**
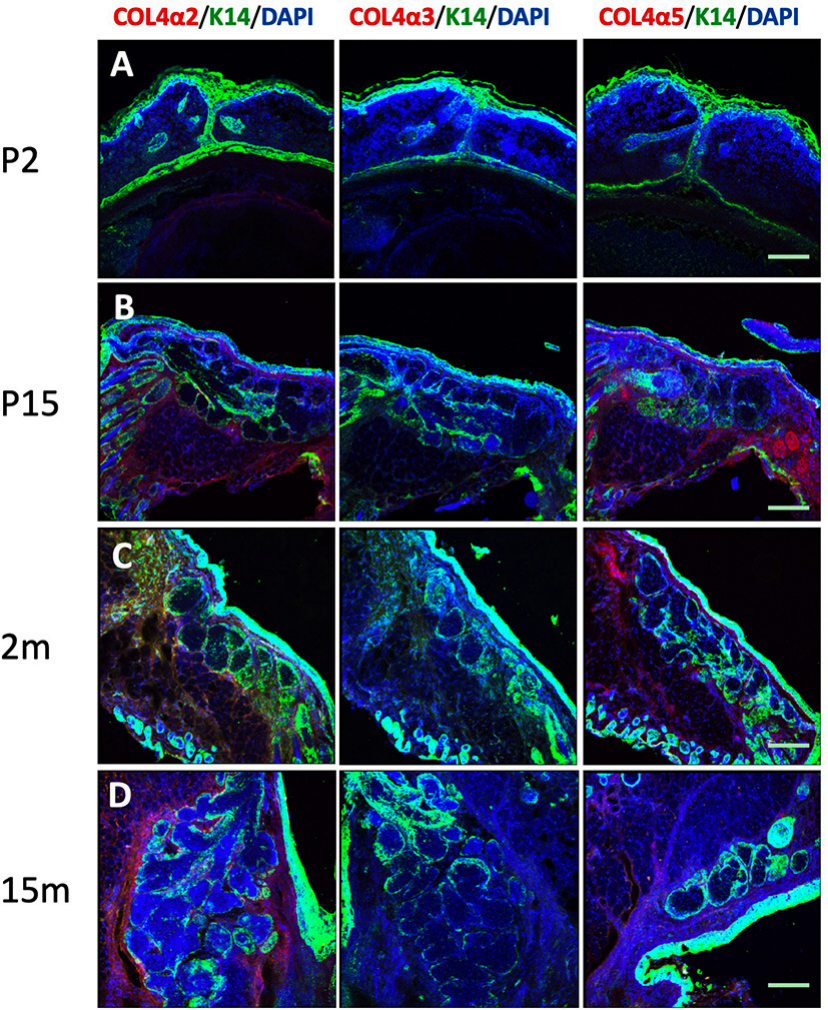
Identification of cytokeratin 14 (K14, green), collagen IV α2 (COL4α2, red), collagen IV α3 (COL4α3, red) and collagen IV α5 (COL4α5, red) in the mouse Meibomian gland of different developmental stages. Nuclei were counterstained with 4', 6-Diamidino-2-phenylindole (DAPI) in blue. **(A)** postnatal day 2 (P2); **(B)** postnatal day 15 (P15); **(C)** 2 months old; and **(D)** 15 months old. Scale bar = 50 μM.

Tissue sections from mice at a range of ages were also stained with the six specific collagen IV subunit antibodies, as well as K14 (to indicate MG epithelial cells) (11) and DAPI (Figure 2). The results show that at postnatal day 2 (P2), when the eyelids are still fused, there is no apparent structure of MG acini or ducts. A solid cord of epithelial cells marked by K14 was visible in the eyelid but staining for all the collagen IV subtypes was negative. By postnatal day 15 (P15), the mature MG structure has developed, with morphology similar to that of adult mice. As in the human tissue sections, four of the six collagen IV subtypes were present around the mature MG; both COL4α3 and COL4α4 were absent. Figure 2 shows the results of staining tissue sections from mice of different ages with COL4α2, COL4α3, and COL4α5. When viewed in combination, these data represent the presence or absence of each of the distinct heterotrimers.

### Glycoproteins

#### Laminins

**Figure 3:**
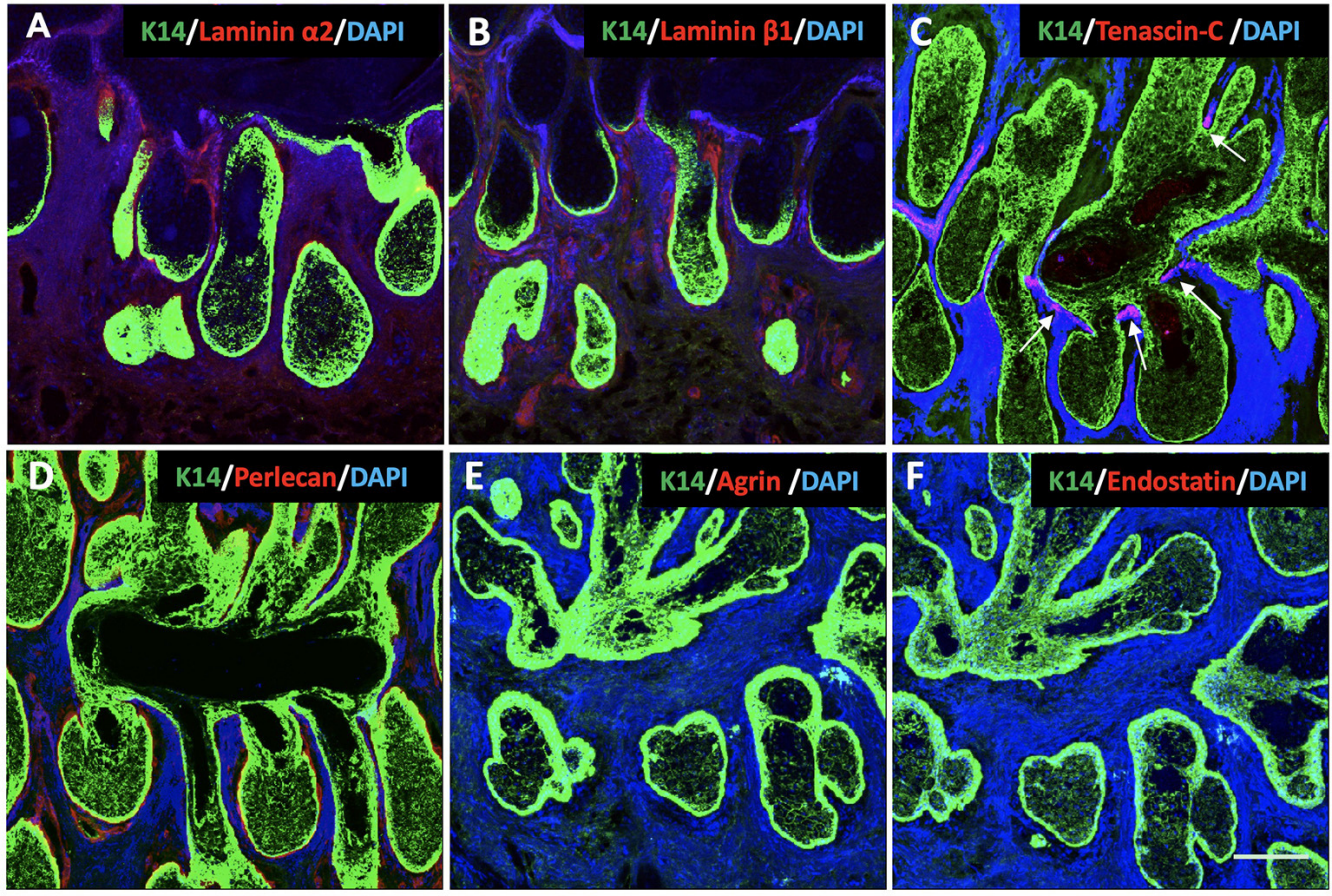
Identification of K14 (green), glycoproteins (red) and proteoglycans (red) in the human Meibomian gland (MG). Nuclei were counterstained with 4', 6-Diamidino-2-phenylindole (DAPI) in blue. Laminin α2 **(A)**, laminin β1 **(B)** and perlecan **(D)** stained positive surrounding the acini and the acinar/ductal junction, but not the region around the duct. Tenascin-C **(C)** was found specifically located at the junctions between the acini and the central duct (white arrows). The staining was negative for both agrin **(E)** and endostatin **(F)** in the human MG tissues. Scale bar = 100 μM.

Laminins are multidomain, heterotrimeric glycoproteins, composed of one each of five α, four β, and three γ chains (20). At least 16 different isoforms have been confirmed in the human body (20). In order to most efficiently identify multiple isoforms, we incubated human MG sections with antibodies specific for laminin α2 and laminin β1. The results show that both the regions surrounding the acini and the acinar/ductal junction, but not the region around the duct, stain positive for laminins (Figures 3A,B).

#### Tenascin-C

Tenascin-C is a large glycoprotein in the ECM that exhibits a very restricted pattern of expression (21). It is an important functional component of various stem cell niches, and plays a major role in regulating stem cell fate (27, 28). Our results show that, in human MGs, tenascin-C is specifically located at the junctions between the acini and the central duct (Figure 3C).

**Proteoglycans**

Proteoglycans are a group of heavily glycosylated molecules found at the periphery of cells. In the BM, these proteoglycans include perlecan, agrin and endostatin (22). In human MG tissue, we observed perlecan around the acini, including at the junction with the duct (Figure 3D). We were unable to identify agrin or endostatin staining in the human tissue sections (Figures 3E,F).

## Discussion

In this study, our data support our hypothesis that the components of ECM are different surrounding the MG acini, ducts and the junctions between them. We have shown that tenascin-C is junction-specific, and that the laminin α2 and β1 chains, as well as perlecan, are located around the acini and junctions. The same collagen IV subtypes are expressed around the entire MG.

A better understanding of the distribution of different ECM components throughout the MG is very important for MG stem cell research. Currently, no specific marker for MG stem cells has been identified. We have previously located MG progenitor cells at the basal layer of the acini (11). Compared to stem cells, progenitors are often unipotent and more specific (29). It is believed that the acinar and the ductal epithelia have distinct progenitor cells. The differences in ECM protein distribution may play a very important role in controlling the activation of MG stem cells to produce more specific progenitor cells. Within the epithelial stem cell niche, stem cells come into direct contact with the ECM, which plays an important role in their maintenance (16). Thus, identifying the major components of the ECM, and drawing a map of their distribution, will increase our understanding of MG physiology and function and, perhaps ultimately, allow us to localize the MG stem cells. Because the MG is a modified sebaceous gland, and there are many similarities between MGs and the sebaceous glands within the pilosebaceous unit (1), we chose to investigate some typical markers surrounding both the sebaceous gland and the hair follicle bulge.

The expression of tenascin-C at the junctions between the MG acini and ducts is very interesting, because it is consistent with the belief that the MG stem cells are located in this region. Our findings are consistent with Milz et al.'s results, which revealed that tenascin was unevenly distributed within the tarsal plate and periglandular areas were often weakly stained (30). The tenascin family is a group of glycoproteins in the ECM (31). While tenascin-C is widespread in embryonic tissues, its expression in adult tissues is restricted to specific sites (31), primarily within stem cell niches (e.g., in the corneal limbus) (28, 32). Tenascin-C has been shown to regulate stem/progenitor cell proliferation and differentiation during organ morphogenesis, turnover and regeneration (28, 33). As the interaction between ECM and stem cells is bidirectional, the splicing, glycosylation and assembly of tenascin-C are regulated by stem cells in the stem cell niche (21). Our observation of tenascin-C differs from findings in other sebaceous glands, possibly due to morphological differences between MGs and hair–associated skin sebaceous glands (28, 34). However, the staining we observed is similar to the localization of tenascin-C within the bulge region of hair follicles, in which the stem cells locate, increasing the likelihood that the MG stem cells will be found at the junctional sites between acini and ducts (34). This situation is also very similar to that of the salivary gland, in which the stem cells are located at the intercalated duct that connects the acinus with the proximal duct (35).

Another major difference in the composition of ECM around MGs is that the laminin α2 and β1 chains and perlecan are only found surrounding the acini and the junctional site. Laminins are the defining component for BM (36). In the epithelial niche, stem cells contact the BM directly, and laminins play a very important role in their maintenance. There are currently 16 different isoforms of laminin identified in the human body: laminins 111 (i.e. α1β1γ1), 121, 211, 213, 221, 311, 312, 321, 332, 411, 421, 422, 423, 511, 521, and 523 (20). Our result shows that at least eight different kinds of laminin may be present in the ECM around MG acini and junctional sites, including laminins 111, 211, 213, 221, 311, 312, 411, and 511. Among these isoforms, laminin 511 has been reported to play a very important role in hair follicle morphogenesis (37). It has also been reported that laminin 511 promotes self-renewal in mouse embryonic stem cells *in vitro* (38). Perlecan is a multifunctional heparan sulfate proteoglycan in the BM. Perlecan participates in a variety of biological activities, including modulating epithelial cell behavior, tissue morphogenesis and metabolism (39–41). It has been reported that perlecan is required for fibroblast growth factor receptor 2 signaling in the neural stem cell niche (42), which is important for meibomian gland homeostasis in the adult mouse (43). Both laminins and perlecan are associated with the regulation of cell quiescence (44, 45). Thus, the distribution of laminin α2 and β1 chains and perlecan may specify the localization of stem and progenitor cells. Combined with the tenascin-C results, we think the location of these markers indicates that the stem cells are located at the junction, while the basal acinar region is likely to be the location of acinar progenitor cells in the MG (8–11).

We did not identify a specific ECM marker located only around the duct. Interestingly, the luminal surface of the duct shows higher staining for K6 than any other part of the duct. In our previous study, we identified K6 as a specific marker for MG ducts (11). K6 has been found in some populations of the luminal ductal cells in other glands (46). A population of K6-positive cells in the prostate gland, which has a high potential for proliferation and differentiation, is a possible candidate for stem cells (47). The pronounced K6 staining in the MG may indicate that this subpopulation of cells comprises the progenitor ductal cells. Because of the similarities between stem and progenitor cells, this may indicate that the previously-suggested “stem cells” at the center of the duct are actually ductal progenitor cells (48, 49).

Collagen IV, a major ECM component, regulates stem cell renewal and tissue regeneration *in vivo* (50). We found that the ECM around the human MG expresses four of the six collagen IV α-chains. Because there are three distinct heterotrimers (α1α1α2, α3α4α5, and α5α5α6), our results indicate that collagen IV in human MG exists in the forms of α1α1α2 and α5α5α6 (19). This result is consistent with observations in the epidermis and sebaceous glands, in which the α3α4α5 chain is also absent (25). We did not observe significant differences in the distribution of the collagen IV subtypes around the MG. This result is similar to previous studies in the salivary gland (51). According to other researchers, the distribution of collagen IV subtypes could change in labial salivary glands under diseased conditions, such as Sjögren Syndrome (35). Because Sjögren Syndrome also impacts MGs and causes MGD (52), it is possible that similar changes could be observed in the MG in Sjögren Syndrome patients. Further studies in this area may help better understand the pathophysiology of MGD.

The results of the mouse tissue samples are also very interesting. The mice at P2 did not have any MG acini or ducts in their fused eyelids; by P15, they showed mature MG morphology. This morphological development process of mice in our study is consistent with the observations of other researchers (53). The mouse samples with mature MG morphology showed collagen IV subtype distribution similar to humans. It appears that the collagen IV does not deposit before the mature MG is formed. Because of the origins of the antibodies, the mouse samples showed high background staining with laminin, tenascin-C, perlecan, agrin and Endostatin antibodies. Thus, we did not draw any conclusion from those data. Future studies with mouse samples are needed. Besides ECM scaffold component, ECM-bound biomolecules are also key component responsible for regulating cells fate (54). Studies in hematopoietic stem cells reported signals from ECM, ECM-bound or diffusible biomolecules could trigger stem cell fate specification events (55, 56). While our study had focused on the scaffold component of ECM, such ECM-bound biomolecules may need future research to identify in the human MG.

In conclusion, our study draws a primary map for the distribution of different components of the ECM around MGs. The discovery of the differences in the ECM surrounding MG structures sheds light on locating the MG stem cells. Because of the close relationship between the ECM and cell fate and function, the identification of the key components in the MG ECM could help us understand the biology and pathophysiology of the tissue. It will also help us to develop *in vitro* culture conditions more closely linked to the *in vivo* state.

## Data availability statement

The original contributions presented in the study are included in the article/supplementary material, further inquiries can be directed to the corresponding author/s.

## Ethics statement

The studies involving human participants were reviewed and approved by Institutional Review Board of the Massachusetts Eye and Ear Infirmary and Schepens Eye Research Institute (SERI). The patients/participants provided their written informed consent to participate in this study. The animal study was reviewed and approved by Institutional Review Board of the Massachusetts Eye and Ear Infirmary and Schepens Eye Research Institute (SERI).

## Author contributions

DC, XC, H-TX, and YL carried out the study. DC drafted the manuscript. DC, XL, and YL conceived the study and its design, as well as revised the manuscript. MH and XL provided the clinical samples. XL and YL supervised and coordinated the study. All authors contributed to the article and approved the submitted version.

## Funding

This research was supported by National Natural Science Foundation of China (Grant No. 82000863), National High Level Hospital Clinical Research Funding (Grant No. 2022-PUMCH-A-198), Young Scholarship Program of Peking Union Medical College Hospital (Grant No. Pumch201910845), NIH Grants (R21028653 and P30EY003790), the Margaret S. Sinon Scholar in Ocular Surface Research Fund, and the David A. Sullivan Laboratory Fund.

## Conflict of interest

Author MH is employed by Ophthalmic Consultants of Boston.

The remaining authors declare that the research was conducted in the absence of any commercial or financial relationships that could be construed as a potential conflict of interest.

## Publisher's note

All claims expressed in this article are solely those of the authors and do not necessarily represent those of their affiliated organizations, or those of the publisher, the editors and the reviewers. Any product that may be evaluated in this article, or claim that may be made by its manufacturer, is not guaranteed or endorsed by the publisher.
